# Multimodal explainable artificial intelligence identifies patients with non-ischaemic cardiomyopathy at risk of lethal ventricular arrhythmias

**DOI:** 10.1038/s41598-024-65357-x

**Published:** 2024-06-27

**Authors:** Maarten Z. H. Kolk, Samuel Ruipérez-Campillo, Cornelis P. Allaart, Arthur A. M. Wilde, Reinoud E. Knops, Sanjiv M. Narayan, Fleur V. Y. Tjong, DEEP RISK investigators, Femke D. Raijmakers, Anne-Lotte C. J. Van Der Lingen, Marco J. W. Götte, Jasper L. Selder, Laura Alvarez-Florez, Ivana Išgum, Erik J. Bekkers

**Affiliations:** 1 Department of Clinical and Experimental Cardiology, Heart Center, Amsterdam UMC Location University of Amsterdam, Meibergdreef 9, Amsterdam, 1105 AZ The Netherlands; 2 Amsterdam Cardiovascular Sciences, Heart Failure & Arrhythmias, Amsterdam, The Netherlands; 3 Department of Medicine and Cardiovascular Institute, Stanford University, Stanford, CA USA; 4 Department of Computer Science (D-INFK), Swiss Federal Institute of Technology (ETH) Zurich, Gloriastrasse 35, Zurich, Switzerland; 5 Department of Cardiology, Amsterdam UMC, Location VU Medical Center, De Boelelaan 1118, Amsterdam, The Netherlands; 6 Department of Biomedical Engineering and Physics, Amsterdam University Medical Center Location University of Amsterdam, Meibergdreef 9, Amsterdam, The Netherlands; 7 Faculty of Science, University of Amsterdam, Science Park 904, Amsterdam, The Netherlands

**Keywords:** Cardiac device therapy, Machine learning

## Abstract

The efficacy of an implantable cardioverter-defibrillator (ICD) in patients with a non-ischaemic cardiomyopathy for primary prevention of sudden cardiac death is increasingly debated. We developed a multimodal deep learning model for arrhythmic risk prediction that integrated late gadolinium enhanced (LGE) cardiac magnetic resonance imaging (MRI), electrocardiography (ECG) and clinical data. Short-axis LGE-MRI scans and 12-lead ECGs were retrospectively collected from a cohort of 289 patients prior to ICD implantation, across two tertiary hospitals. A residual variational autoencoder was developed to extract physiological features from LGE-MRI and ECG, and used as inputs for a machine learning model (DEEP RISK) to predict malignant ventricular arrhythmia onset. In the validation cohort, the multimodal DEEP RISK model predicted malignant ventricular arrhythmias with an area under the receiver operating characteristic curve (AUROC) of 0.84 (95% confidence interval (CI) 0.71–0.96), a sensitivity of 0.98 (95% CI 0.75–1.00) and a specificity of 0.73 (95% CI 0.58–0.97). The models trained on individual modalities exhibited lower AUROC values compared to DEEP RISK [MRI branch: 0.80 (95% CI 0.65–0.94), ECG branch: 0.54 (95% CI 0.26–0.82), Clinical branch: 0.64 (95% CI 0.39–0.87)]. These results suggest that a multimodal model achieves high prognostic accuracy in predicting ventricular arrhythmias in a cohort of patients with non-ischaemic systolic heart failure, using data collected prior to ICD implantation.

## Introduction

**Figure 1:**
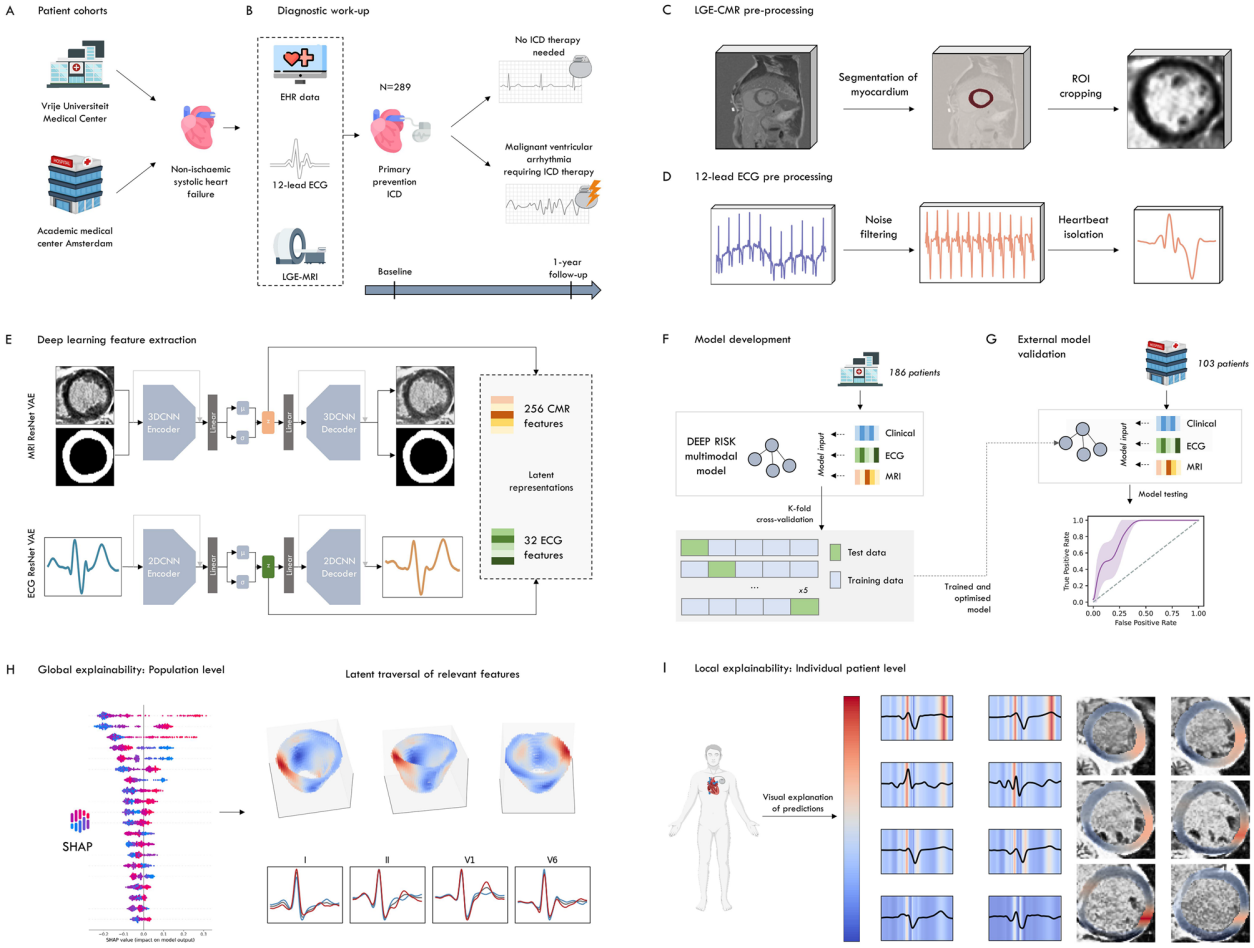
Overview of the study workflow. (**A**) Patient with a non-ischaemic cardiomyopathy were included from two academic hospitals in The Netherlands. (**B**) The raw late gadolinium enhancement magnetic resonance imaging (LGE-MRI) scans, 12-lead electrocardiograms (ECGs), and clinical patient information were collected. Occurrences of malignant ventricular arrhythmia within 12 months from ICD implantation were retrieved. (**C**,**D**) Pre-processing of the ECG and LGE-MRI data, including myocardium segmentation, region-of-interest cropping and mean waveform isolation. (**E**) Variational autoencoders extracted the latent representations from MRI (256 latent representations) and ECG (32 latent representations). (**F**,**G**) Supervised machine learning models were trained to predict the outcome in the development cohort, and validated in the external patient cohort. (**H**) Latent traversal depicted the global explainability of latent representations, gradient-based activations map provided patient-level explainability.

Implantable cardioverter-defibrillators (ICDs) play a crucial role in the prevention of sudden cardiac death (SCD) caused by malignant ventricular arrhythmias^1^. However, the efficacy of ICD treatment is increasingly debated among patients with non-ischaemic causes of systolic heart failure^1–3^. Although early studies demonstrated survival benefit of a prophylactic ICD in patients with non-ischaemic systolic heart failure (NICM)^4–7^, there is recent concern that these patients may receive modest benefit from the ICD^2,8^. The sensitivity of left ventricular ejection fraction to predict SCD is poor, which necessitates a more refined approach of risk stratification that selects patients at highest risk for SCD and balances the risks and benefits from prophylactic ICD implantation^9^. In particular, the presence of myocardial fibrosis is strongly related to arrhythmic events in NICM patients with mild left ventricular dysfunction, therefore not considered eligible for ICD implantation^10,11^. Improved arrhythmic risk prediction may be achieved through comprehensive modelling of electrophysiological mechanisms and myocardial substrate characteristics using neural networks, that can learn non-linearities and complex patterns from high-dimensional data^12,13^. In relatively homogeneous cohorts of patients with an ischaemic cardiomyopathy, neural networks have been shown to capture high-level representations from cardiac magnetic resonance imaging (CMR) and electrophysiological signals, that reflect ventricular arrhythmia risk^14–16^. This approach could prove beneficial in non-ischaemic cardiomyopathy, which encompasses heterogeneous inflammatory, infiltrative and genetic pathologies, each of which likely has a distinct SCD risk profile^9^. As of yet, it is unexplored whether a deep learning approach may extract predictive signatures for ventricular arrhythmia in patients with diverse non-ischaemic substrates for cardiomyopathy. In this study, we developed and externally validated an explainable multimodal deep learning pipeline that predicted ventricular arrhythmia risk in patients with non-ischaemic systolic heart failure in the first year after ICD implantation (Fig. 1).

## Methods

### Ethics

The study was approved by the Institutional Review Boards of the Amsterdam University Medical Center (*Medisch Ethische Toetsingscommissie AMC*, date 29-04-2021, approval number 21.230) and Vrije Universiteit Medical Center (*Medisch Ethische Toetsingscommissie VUmc*, date 26-06-2020, approval number 2020.296). The requirement for written informed consent was waived as the medical research involving Human Subjects Act did not apply.

### Subjects

Patients with non-ischaemic systolic heart failure (left ventricular ejection fraction ≤ 45%), implanted with a de novo ICD for primary prevention of SCD were eligible. Patients were included if they had: (i) at least one MRI with late gadolinium enhancement (LGE) and 12-lead ECG within 5 years before ICD implantation; (ii) available clinical baseline information and medication status; (iii) minimum follow-up duration of at least one year from ICD implantation onwards. Patient data was retrospectively collected from two academic, tertiary hospitals in Amsterdam, The Netherlands. Patients were implanted with an ICD between 2007 and 2021. This study adheres to the reporting guidelines for Transparent Reporting of a multivariable prediction model for Individual Prognosis or Diagnosis (TRIPOD), where applicable^17^.

### Outcome of interest

The outcome of interest was any malignant ventricular arrhythmia during the first year after ICD implantation, defined as an episode of sustained ventricular tachycardia or ventricular fibrillation, treated by the ICD through a shock and/or anti-tachycardia pacing (ATP). Outcomes were collected from electronic health records (EHR), and adjudicated by the clinicians.

### Clinical data

At both sites, demographic data, medical history, laboratory values, body mass index (BMI) and medication status (i.e. anti-arrhythmic, anti-coagulant, anti-hypertensive, and lipid-lowering drugs) were retrieved from the EHR. Missing values were imputed using a non-parametric imputation method based on random forests (*missForest* package, version 1.1.3. in Python)^18^. Variables with over 30% missing values were excluded (Supplementary Table 1 shows the percentages of missing data). Categorical variables were one-hot encoded, continuous variables were standardised using z-scores.

### MRI retrieval and pre-processing

Short-axis raw LGE-MRI phase-sensitive inversion recovery scans were retrieved from the systems, and identified using DICOM tags (Supplementary Methods p1). The median number of days between LGE-CMR and ICD implantation was 77 days [interquartile range (IQR) 19–211]. First, we used a pre-trained convolutional neural network for segmenting the myocardium (Fig. 1C). This segmentation model was based on a U-net architecture^19^, characterised by four down-sampling steps using max-pooling in the encoder section, and four upsampling steps through deconvolution in the decoder layers. Subsequently, the pixel-wise probability maps for the myocardium were used to crop the images around the region of interest^20^. The cropped images were resized for uniformity, resulting in each set comprising 12 slices of height and width of 64 pixels. Histogram equalisation was used to improve contrast in images. Next, histogram normalisation was applied to standardise their intensity values. After the myocardium masks were adjusted by zeroing out regions outside the myocardium wall, these were layered over the pre-processed LGE-MRIs, resulting in images dimensions of 12 × 64 × 64 × 2 (slices × height × width × myocardium mask and scan). The spatial resolutions of these images varied, with heights and widths ranging from 1.5 to 2.4 mm and a depth of 10 mm.

### ECG retrieval and pre-processing

Raw-format 12-lead 10-s resting ECGs were collected retrospectively at both sites (Supplementary Methods p1). After downsampling to 250 Hz, raw signals underwent noise filtering and baseline wander removal using a Savitzky-Golay Filter for smoothing via high-order polynomial fitting and a low-resolution Fourier series subtraction for eliminating baseline wander^16,21^. Individual heartbeats were isolated for each ECG lead by automatic marking of individual R-peak locations, and subsequent extraction of heartbeat templates given a list of these locations (Fig. 1D)^16^. Individual P-QRS-T segments were aligned, after which mean waveforms were calculated by averaging individual waveforms per unique lead. ECG waveforms were pre-processed by normalising the signals between 0 and 1 using z-score.

### Residual variational autoencoder architecture

We employed a β-VAE neural network architecture for feature extraction from ECGs and LGE-MRI SAX images^22–24^. Unlike traditional autoencoders, β-VAE introduces a probabilistic element, enabling the model to learn a probabilistic mapping between input data and a lower-dimensional latent space (Supplementary Methods p.2,3). These architectures (Supplementary Fig. 1) included convolutional layers in the encoder for spatial pattern capture from the input data, and upsampling in the decoder to transform features sampled from the latent space back to the original data domain. Residual blocks were used to facilitate identity mapping for efficient information propagation. The β-VAEs optimized the Evidence Lower Bound (ELBO), consisting of expected log-likelihood (in this case reconstruction loss) and β-weighted Kullback–Leibler divergence (which induced latent space regularization) (Supplementary Methods p.2)^22,23^. The β parameter crucially balances disentanglement and reconstruction accuracy^24^. We tuned β for optimal performance, trained models on ECGs and LGE-MRIs, and evaluated their reconstructive performances (Supplementary Figs. 3, 4, Supplementary Methods p.3,4)^24^. We trained and validated both VAE networks on larger datasets, after which the weights of the optimised model were used to obtain the latent representations (Supplementary Method p.3, Supplementary Fig. 2). The 2D-ECG VAE was trained and validated on 333,304 12-lead ECG waveforms, encoding these to a latent space dimensionality of 32. The 3D-MRI VAE was trained and validated on 970 scans, encoding to a latent space dimension of 256. Reconstructive performances of both networks were assessed by comparing original model input to the decoded output (Supplementary Methods p.3,4). The ResNet VAEs were implemented using PyTorch (version 2.0.5) for Python (version 3.11.4).

### Training and external validation of the prediction model

Supervised machine learning models using Extreme Gradient Boosting (XGBoost) were trained and optimised on latent representations obtained from the LGE-MRI scans and 12-lead ECG mean waveforms, and clinical patient data, to predict the one-year probability of a malignant ventricular arrhythmia^25^. Alternative models were trained exclusively on latent representations derived from either LGE-MRI or ECG, or clinical patient data. Supplementary Table 3 provides a list of the clinical variables used as input. Bayesian optimisation was used to tune the hyperparameters of each model, using a stratified k-fold cross-validation strategy. The hyperparameter search space is displayed in Supplementary Table 2. We used synthetic minority over-sampling technique (SMOTE) to address class imbalance in the dataset by generating synthetic examples of the minority class during model development^26^. Following hyperparameter optimisation, the best performing model was re-trained on all patients in the development cohort (Hospital A) and evaluated in the external patient cohort (Hospital B).

### Model explainability

We aimed to explain the model’s behaviour through a two-step approach. First, the output of the prediction model was explained by SHapley Additive exPlanations (SHAP)^27^. SHAP, rooted in the Shapley value concept from game theory, assigns a feature importance value to each element in a prediction, where a positive SHAP value indicates that a feature contributed to increasing the probability of the prediction, while negative values suggest a negative impact on the predicted probability. This method allows for explainability on a global (population) and local (patient) level. Features with highest impact on the predicted outputs were subsequent explored using a latent traversal procedure (global explainability) and gradient-based activation maps (local explainability). In brief, latent traversal involved a systematic alteration of the latent vector (the learned representation of the input data in the latent space) at a single instance, subsequently feeding this changed latent vector to the decoder. Next, generated reconstructions from the altered latent vector were assessed for semantically meaningful transformations (Supplementary Methods, p. 4). On a patient level, we aimed to provide a physiological explanation of relevant latent representations through gradient-based attention mapping. In contrast to classification models that compute attention maps by backpropagating the prediction outcome, we used the learned latent space directly to generate the attention maps (Supplementary Methods, p. 4,5)^28^. Gradient-based attention maps were generated and averaged for the ten latent representations with the highest SHAP importances for each patient. This process resulted in patient-specific heatmaps, which were then superimposed onto the original MRI scans and ECGs, highlighting regions of interest.

### Statistical analysis

Continuous variables were described using either the median and IQR or the mean and standard deviation, depending on their distribution. Categorical sociodemographic and clinical variables were summarised as frequencies and percentages. Baseline characteristics were presented for both patient cohorts. Performance of the classifier was assessed on the external validation cohort, using a bootstrapping technique involving 3000 iterations with resampling to calculate 95% confidence intervals (CI) around the performance metrics. Model performances were visualised using receiver operating characteristic (ROC) curves and precision-recall (PR) curves, and were assessed based on metrics including the area under the ROC curve (AUROC), sensitivity, specificity, positive predictive value, negative predictive value and accuracy. AUROCs were compared using DeLong’s test. F1-scores and area under the precision recall curve (AUPRC) were calculated as a more robust measures to class-imbalance. The Youden's J statistic was used to determine the optimal cut-point, optimising the balance between sensitivity and specificity. Model calibration was assessed visually and assessed by the slope of the calibration curve. All statistical analyses were performed using Python (version 3.11.4).

### Role of the funding source

The funding source had no role in the study design, data collection, data analyses, interpretation, or writing of report.

## Results

The CONSORT diagram in Supplemental Fig. 5 outlines the selection of our study cohorts. A summary of the cohort statistics was provided in Table 1. A total of 289 NICM patients were enrolled. The development cohort consisted of 186 ICD recipients; the external testing cohort consisted of 103 patients. The mean age across cohorts was 59.6 ± 14.5 years, and 93 patients (32.2%) were female. The aetiology of heart failure was dilated cardiomyopathy in the majority of patients (63.3%), 97 (33.6%) patients received a CRT-D. In total, 26 (9%) patients experienced a malignant ventricular arrhythmia treated by the ICD in the first year after device implantation. This comprised 22 subjects who received a shock and/or ATP, and four subjects received ATP only. During a one year follow-up period, nine (4.8%) patients in the development cohort and one (1.0%) patient in the validation cohort died.

**Table 1:** Baseline characteristics for the development cohort and the external validation cohort.

	Development cohort (n = 186)	External validation (n = 103)	*p*-value
Age, mean (± SD)	63.0 (12.5)	53.3 (15.7)	< 0.001
Female, yes (%)	68 (36.6)	25 (24.3)	0.044
BMI (kg/m^2^), mean (± SD)	26.8 (4.8)	25.5 (4.5)	0.035
Underlying pathology, yes (%)			< 0.001
Dilated	141 (75.8)	42 (40.8)	
Genetic	11 (5.9)	4 (3.9)	
Hypertrophic	18 (9.7)	27 (26.2)	
Miscellaneous	16 (8.6)	30 (29.1)	
Medical history, yes (%)			
Atrial arrhythmia	49 (26.3)	31 (30.1)	0.585
CVA	16 (8.6)	9 (8.7)	1.000
COPD	20 (10.8)	5 (4.9)	0.136
Hypertension	90 (48.4)	35 (34.0)	0.025
Diabetes mellitus	35 (18.8)	13 (12.6)	0.234
Prior non-sustained VT	49 (26.3)	32 (31.1)	0.287
Laboratory, mean (± SD)			
Sodium, mmol/L	139.8 (3.2)	139.9 (2.5)	0.605
Potassium, mmol/L	4.4 (0.5)	4.3 (0.4)	0.063
Creatinine, µmol/L	97.5 (50.1)	112.2 (106.4)	0.132
Medication, yes (%)			
β-blocker	156 (83.9)	56 (63.6)	< 0.001
ARB/ACEi	178 (95.7)	96 (93.2)	0.148
Vitamin K antagonist	53 (28.5)	14 (16.1)	0.039
Sotalol	6 (3.2)	4 (4.6)	0.731
Digoxin	16 (8.6)	7 (8.0)	1.000
Amiodarone	11 (5.9)	10 (11.5)	0.171
NOAC	8 (4.3)	15 (17.2)	0.001
Device, yes (n%)			< 0.001
Single-chamber	55 (29.6)	30 (29.1)	
Dual-chamber	50 (26.9)	21 (20.4)	
CRT-D	74 (39.8)	23 (22.3)	
Subcutaneous ICD	7 (3.8)	29 (28.2)	

*ACEi* angiotensin-converting enzyme inhibitors, *ARB* angiotensin II receptor blocker medication, *BMI* body mass index, *COPD* chronic obstructive pulmonary disease, *CRT-D* cardiac resynchronization therapy with defibrillator, *CVA* cerebrovascular accident, *NOAC* new oral anticoagulants, *VT* ventricular tachycardia.

### Reconstruction performance of ResNet VAEs

The 2D ResNet ECG-VAE reconstructed mean 12-lead ECG waveforms with a mean Pearson correlation coefficient of 0.97 ± 0.005 and a mean root mean squared error of 0.04 ± 0.003. MRI-LGE scans were reconstructed with a structural similarity index measure (SSIM) of 0.39 ± 0.09. A complete overview of the reconstructive performances is provided in Supplementary Tables 4–5, example reconstructions are displayed in Supplementary Figs. 6, 7.

### Predictive performance of the latent variables

**Figure 2:**
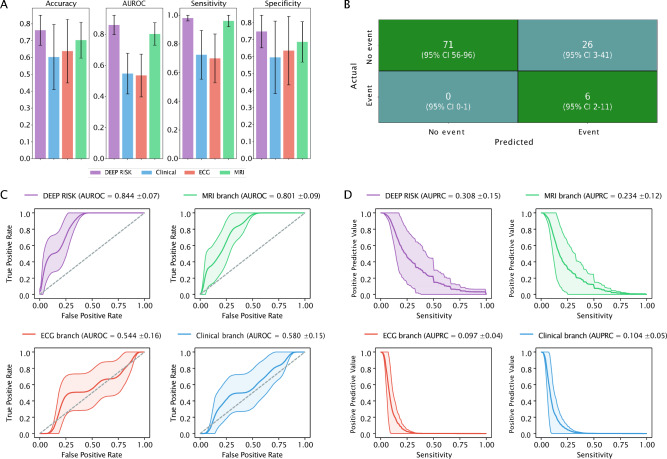
Model performance on an external patient cohort. (**A**) Bar plots of the performance metrics across models. (**B**) Presents the confusion matrix of the predicted vs. actual events of the classification model. Mean values were averaged over the total number of bootstrap iterations. (**C**) Receiver operating characteristic curves for each model. (**D**) Shows the precision recall curves. Area under the receiver operating characteristic curve and area under the precision-recall curve are displayed for the multimodal DEEP RISK model and alternative models trained a single domain. *AUROC* area under the receiver operating characteristic curve, *AUPRC* area under the precision-recall curve.

Performance metrics of the multimodal DEEP RISK prediction model tested in the external patient cohort are displayed in Table 2. Figure 2 visualises the performance of DEEP RISK, compared to the alternative models trained on data from one modality only (MRI, ECG and clinical branches). DEEP RISK reached an AUROC of 0.84 (95% 0.71–0.96) and an AUPRC of 0.31 (95% CI 0.08–0.65). At the optimal cut-point, the model predicted malignant ventricular arrhythmias with 98.1% (95% CI 75.0–100%) sensitivity and 72.6% (95% CI 58.0–96.9%) specificity. This corresponds to a 99.9% (95% CI 98.6–100.0%) probability of a patient having no malignant ventricular arrhythmia, given a negative prediction. Calibration curve visualisation (Supplementary Fig. 8) showed overestimated SCD risk prediction. The MRI branch reached good discriminative performance (AUROC 0.80, 95% CI 0.65–0.94), while the ECG (AUROC 0.64, 95% CI 0.39–0.87) and clinical (AUROC 0.54, 95% CI 0.26–0.82) branches performed poorly in the external cohort (DeLong’s p-values < 0.001 for comparison with the MRI branch). The performance of DEEP RISK was higher compared to the MRI branch (DeLong’s p-value < 0.001). Figure 2B displays the average number of false vs. true predictions in the external cohort, ROC and PR curves are displayed in Fig. 2C,D. A comprehensive overview of performance metrics is provided in Supplementary Table 6.

**Table 2:** Performance of risk prediction in an external patient cohort.

	DEEP RISK	Clinical branch	ECG branch	MRI branch
Accuracy	0.741 (0.602–0.961)	0.625 (0.214–0.922)	0.643 (0.136–0.864)	0.706 (0.515–0.951)
Sensitivity	0.981 (0.750–1.000)	0.819 (0.364–1.000)	0.703 (0.333–1.000)	0.955 (0.714–1.000)
Specificity	0.726 (0.580–0.969)	0.613 (0.172–0.938)	0.641 (0.091–0.878)	0.690 (0.495–0.96)
AUROC	0.844 (0.713–0.961)	0.639 (0.386–0.867)	0.544 (0.260–0.816)	0.801 (0.649–0.936)
AUPRC	0.313 (0.083–0.652)	0.131 (0.035–0.294)	0.099 (0.033–0.206)	0.232 (0.061–0.499)

*AUROC* area under the receiver operating characteristic curve, *AUPRC* area under the precision-recall curve, *ECG* electrocardiogram, *MRI* magnetic resonance imaging, *PPV* positive predictive value, *NPV* negative predictive value.

### Explainability of predictions

**Figure 3:**
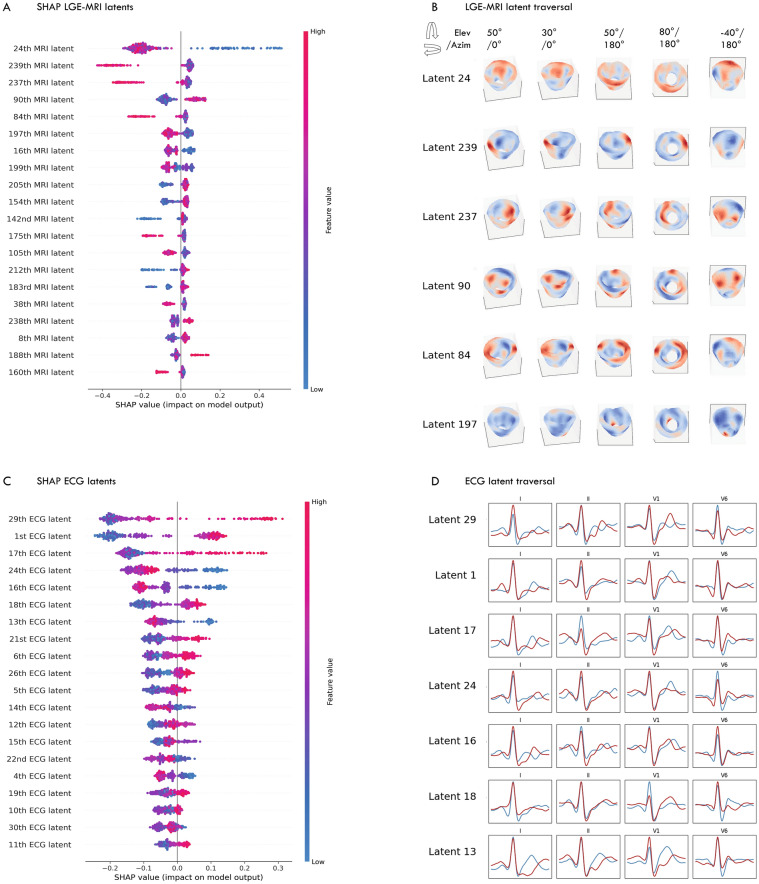
Global explainability of the latent representations. (**A–C**) SHAP values for the LGE-MRI and ECG latent representations. (**B–D**) Latent traversal depicting semantically relevant transformations in LGE-MRI and ECG. Latent variables were systematically changed from − 3 until + 3 standard deviations, relative to the mean value.

**Figure 4:**
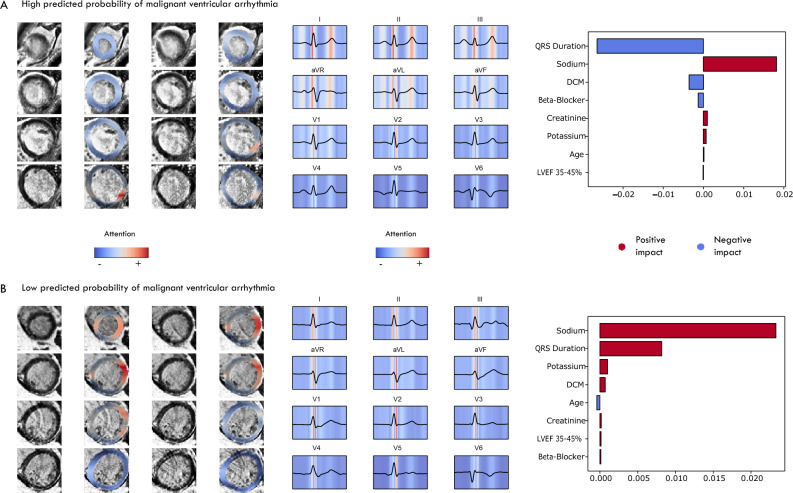
Gradient-based activation maps visualise the importance of regions in the LGE-MRI and ECG. *LGE-MRI* late gadolinium-enhanced magnetic resonance imaging, *ECG* electrocardiogram.

Figure 3A displays the latent MRI representations with the highest feature importance as provided by SHAP, while Fig. 3B illustrates the semantic transformations resulting from the traversal of a specific latent. Latent variables varied in representing changes in myocardium characteristics, exhibiting focal differences (e.g., apical region vs. basal regions) as well as more scattered or global transformations. Figure 3C,D illustrate these transformations on the ECG morphology. Figure 4A and B display the regions in the myocardium and in the 12-lead ECG that affected the predicted probability of ventricular arrhythmia in two patients from the external patient cohort. Regions in the myocardium that exhibit late enhancement, along with non-enhanced regions that might otherwise have gone unidentified, have influenced the predictions. Latent representations reflecting the QRS morphology, and to a lesser extent, the T-wave in leads V2-V6, were found to be relevant in the context of arrhythmic risk.

## Discussion

The efficacy of primary prevention ICD treatment in patients with a non-ischaemic cardiomyopathy (NICM) is questioned. In a contemporary population of patients with NICM, the DANISH (Danish Study to Assess the Efficacy of ICDs in Patients with Non-ischemic Systolic Heart Failure on Mortality) randomised trial demonstrated no difference in mortality rates between NICM patients assigned to receive an ICD, and those undergoing standard clinical care^2^. In addition, in the extended follow-up of the SCD-HeFT trial, it was observed that mortality rates in patients with ICDs were similar when compared with those who received a placebo^8^. As a result, the guideline recommendations for ICD patient selection have been changed from class I to IIa, affecting the rate of prophylactic ICD implantations in patients with NICM^1,29^. Conversely, prior analyses have demonstrated various subsets of patients, such as those under 70 years, to experience benefit from an ICD^30,31^. In other words, a uniform strategy for SCD prevention in a NICM population that fails to account for the heterogeneity between patients could potentially lead to improper withholding of an ICD. This ambiguity underscores the immediate need for tools that allow for a personalised risk-stratification, that could guide clinicians during patient selection for prophylactic ICD implantation^9^.

**Figure 5:**
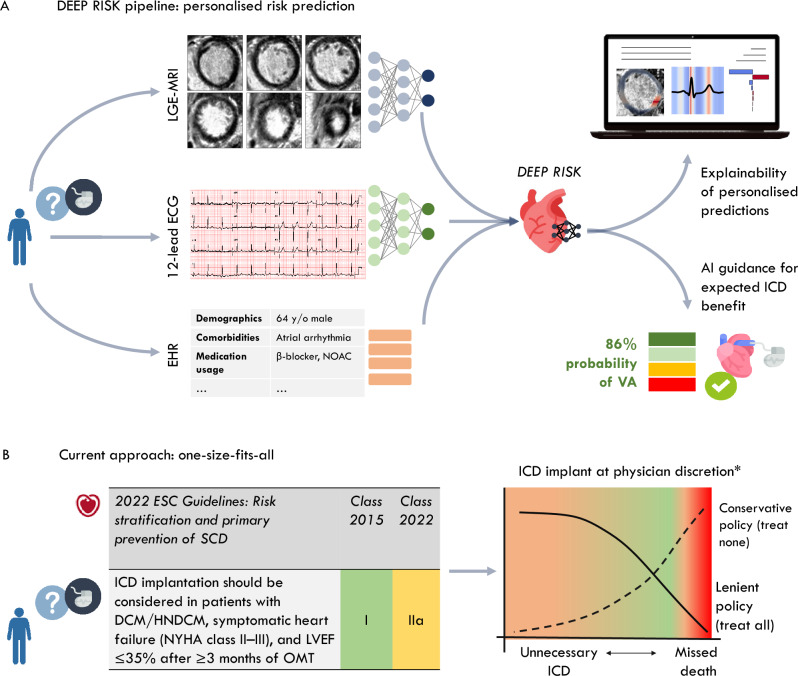
Proposed workflow that incorporates the DEEP RISK model for an individual patient. (**A**) Raw LGE-MRI and 12-lead ECG are required as input to neural networks that extract the latent representations. These enrich the clinical patient information from electronic health records, providing multimodal data to the DEEP RIKS algorithm that provides a personalised risk score combined with an explanation of model decisions. (**B**) Current risk stratification guides clinicians through a one-size-fits-all recommendation. Depending on a lenient (implant all) vs. conservative (implant none) policy, this leads to unnecessary ICD implantations or missed deaths, respectively. *The National Health Care Institute in The Netherlands advises against using ICDs in patients with non-ischemic cardiomyopathy. *LGE MRI* late gadolinium-enhanced magnetic resonance imaging, *ECG* electrocardiogram.

In this study, we have demonstrated that neural networks were able to extract the relevant high-level physiological representations from LGE-MRI scans and 12-lead ECG, which, integrated with clinical patient information, could be leveraged for accurate personalised arrhythmic risk prediction. At external model validation in a cohort of 103 patients, DEEP RISK on average correctly identified the six malignant ventricular arrhythmias and 71 correct (at least yearly) withholdings of ICD. This came at the expense of 26 ‘unnecessary’ implantations and no missed malignant ventricular arrhythmia (and thus potential preventable arrhythmic death). Despite important differences in patient mix between the development and validation cohorts, such as the underlying causes of NICM and percentage of patients with CRT, the model reached an AUROC of 0.84 (95% CI 0.71–0.96). In particular, the prognostic information within LGE-MRI and the capacity of the ResNet VAE to derive this information from the scan, was evident in the gap observed in predictive accuracy between this branch (AUROC 0.80), compared to clinical and ECG branches (respectively AUROC of 0.64 and 0.54). Myocardial fibrosis is recognised as a substrate for the development of ventricular arrhythmias, however, the prognostic role of LGE-MRI in arrhythmic risk-stratification for patients with NICM is still under debate^32–34^. Prior observational studies have demonstrated increased risk of ventricular arrhythmia in the presence of myocardial fibrosis in patients with NICM^10,35–37^. Nevertheless, the DANISH substudy demonstrated that ICD implantation did not reduce the risk of all-cause death, regardless of the presence of LGE^38^. The presence of LGE, often treated as a binary factor in these studies, may simplify the complex nature of late-enhanced regions and overlook the tissue characteristics and spatial distributions that reflect arrhythmic risk. Data-characterisation algorithms applied to LGE-MRI have demonstrated the ability to extract 'hand-crafted' features from the enhanced regions with prognostic capability^39–41^. Alternatively, neural networks, adept at learning hierarchical representations and capturing intricate patterns within complex data, are particularly powerful at learning abstract features directly from the data^14^. In this work, we are the first to show the capacity of deep neural networks to extract meaningful information from both LGE-MRI and ECG in an NICM patient population, that could significantly contribute to arrhythmic risk stratification. Figure 5 outlines the envisioned use of DEEP RISK as a point-of-care prediction model, compared to the current guideline recommendations, to guide the timing of ICD implantation.

Crucial for achieving this level of applications of the DEEP RISK framework are model explainability and robustness. Latent traversal, which visualises the semantic representation of an individual latent variable, indicated both focal areas and larger regions of enhancement to modulate the predicted probability of malignant ventricular arrhythmia onset. This global explainability provides an insight in the model’s decision-making process in relation to all its inputs, but this does not necessarily explain predictions for an individual patient. Our aim was to offer this level of local explainability through gradient-based activation heatmaps, which visualise regions in the myocardium relevant to predicting ventricular arrhythmias. Interestingly, these visualisations demonstrated prognostic information in both enhanced regions, as well as regions without late enhancement. This is in line with prior studies that used deep neural networks to learn the relevant features from cardiac MRI, and use these for accurate arrhythmia prediction^14,42^. Other than the LGE-MRI latent representation capturing important prognostic information, there was incremental prognostic value in ECG and clinical patient information. This suggests that the deep learning features obtained from LGE-MRI and ECG provided complementary physiological information, potentially reflecting both proarrhythmic substrate and triggering mechanisms^43^. Although several studies have demonstrated information from 12-lead ECG to be sufficient for accurate personalised arrhythmia prediction, we observed that the discriminative ability of latent ECG representations in a NICM patient population was poor^12^. A recent study that used an ECG-AI index for predicting SCD in patients with a NICM reached an AUROC of 0.68 (95% CI 0.59–0.77)^44^. In addition, a dynamic prediction model for ventricular arrhythmia onset that leveraged routinely collected longitudinal ECG recordings reached an AUROC 0.74 ± 0.07^16^. Arguably, the snapshot mean waveforms used as input to the ResNet VAEs were insufficient to fully capture the electrophysiological instabilities that increase the risk of ventricular arrhythmias, such as changes in the autonomic nervous system^45^. As such, a promising opportunity for further exploration is to learn a shared latent space across different domains, enabling more holistic latent representations^46,47^.

### Limitations

The major limitation of this study is the known discrepancy between appropriate ICD-therapy for a malignant ventricular arrhythmia and actual SCD. Ventricular arrhythmic events triggering ICD-therapy might have naturally self-terminated, leading to an overestimation of the proportion of patients who truly benefit from the ICD^48^. Second, a higher proportion of ICD recipients underwent LGE-MRI at Hospital B, as compared to Hospital A, which may have introduced a selection bias and may affect the generalisability of the current model. Furthermore, machine learning models are prone to selection bias when the training data does not accurately reflect the diversity and characteristics of the population being modelled. Therefore, it is imperative to evaluate the predictive performance across different groups within the broader NICM population in a prospective study. Third, gradient-based attention mapping, effective in identifying critical input regions, may not fully capture complex spatial relationships essential in medical imaging, potentially oversimplifying critical features. Its reliance on gradients from the last convolutional layer might not detect subtle yet significant patterns crucial for clinical interpretations. Moreover, the disentanglement of latent variables, although crucial for understanding real-world significance, may not have a straightforward clinical interpretation, posing challenges in translating model findings to practical medical insights^49^. From a methodological perspective, there is a need for studies to explore the physiological mechanisms underlying prognostic latent representations inferred from the LGE-CMR, and their relation to human-interpretable risk factors such as LGE presence and scar mass. Fourth, it is crucial to test the predictive performance of the model across various underlying causes of non-ischaemic cardiomyopathy, larger cohorts of CRT patients, and patients using novel heart failure medications.

## Conclusion

We demonstrate that neural networks are able to extract relevant high-level representations from LGE-MRI images and ECG, that can be leveraged for accurate personalised prediction of malignant ventricular arrhythmias in patients with non-ischaemic systolic heart failure. We explored the learned latent spaces to provide global (population level) and local (individual patient level) explainability, providing the potential physiological underpinnings of predictions. With further corroboration, a multimodal computational approach may facilitate point-of-care arrhythmic risk stratification, enabling clinicians to identify NICM patients who would benefit from an ICD.

### Supplementary Information

Supplementary Information.

## Data Availability

Data sharing requests will be considered upon a reasonable request. For access, please email the corresponding author. Code scripts are available at: https://github.com/DeepRiskAUMC/DEEPRISK-multimodal-model.
