# Supplementary material for: Multimodal explainable artificial intelligence identifies patients with non-ischaemic cardiomyopathy at risk of lethal ventricular arrhythmias
Source: Sci Rep. 2024 Jun 27;14:14889. doi: 10.1038/s41598-024-65357-x (PMC11211323; doi:10.1038/s41598-024-65357-x)
Supplement: Supplementary file 1 — Supplementary Information. [file 41598_2024_65357_MOESM1_ESM.docx]

**Appendix**

[Supplementary Methods 1](#_Toc157518022)

[**CMR acquisition protocol and parsing of DICOM files** 1](#_Toc157518023)

[**Residual Variational Autoencoder Architecture** 1](#_Toc157518024)

[**β parameter tuning** 2](#_Toc157518025)

[**Model training** 3](#_Toc157518026)

[**Reconstructive performance assessment** 3](#_Toc157518027)

[**Extreme Gradient Boosting Algortihm (XGBoost) developement** 4](#_Toc157518028)

[**Latent representation traversal** 4](#_Toc157518029)

[**Gradient-based activation mapping** 4](#_Toc157518030)

[Supplementary Tables 5](#_Toc157518031)

[**Supplementary table 1.** Percentages of missing values in the clinical patient data 5](#_Toc157518032)

[**Supplementary table 2.** Search space used for tuning of hyperparameters of the extreme gradient boosting algorithm (XGBoost) 6](#_Toc157518033)

[**Supplementary table 3.** Clinical variables used for predictive modelling 7](#_Toc157518034)

[**Supplementary table 4.** Reconstruction performances of the 3D ResNet VAE 8](#_Toc157518035)

[**Supplementary table 5.** Reconstruction performances of the 2D ResNet VAE for ECG reconstruction 9](#_Toc157518036)

[**Supplementary table 6.** Performance on the external validation test for one-year appropriate ICD-therapy risk prediction 10](#_Toc157518037)

[Supplementary Figures 11](#_Toc157518038)

[**Supplementary figure 1.** Schematic overview of the ResNet VAEs for MRI (3D) and ECG (2D) reconstruction 11](#_Toc157518039)

[**Supplementary figure 2.** Model development and extraction of the latent representations from patients in the NICM cohort 11](#_Toc157518040)

[**Supplementary figure 3.** Optimisation of the β-parameter of the ResNet variational autoencoder (VAE) for 12-lead ECG reconstruction 12](#_Toc157518041)

[**Supplementary figure 4.** Optimisation of the β-parameter of the ResNet variational autoencoder (VAE) for short-axis LGE-MRI reconstruction 12](#_Toc157518042)

[**Supplementary figure 5.** CONSORT diagram of patient selection 13](#_Toc157518043)

[**Supplementary figure 6.** Original and reconstructed mean 12-lead ECG waveforms 14](#_Toc157518044)

[**Supplementary figure 7.** Original and reconstructed short-axis LGE-MRI images 14](#_Toc157518045)

[**Supplementary figure 8.** Calibration curve for the multimodal prediction model 15](#_Toc157518046)

[**References** 16](#_Toc157518047)

# Supplementary Methods

**CMR acquisition protocol and parsing of DICOM files**

At both sites, a clinical 1.5-T MRI scanner (Magnetom Avanto, Siemens, Erlangen, Germany) was used with a dedicated phased-array body coil. Standard 4-, 3- and 2-chamber orientations were obtained, and subsequently a stack of 10 to 12 consecutive short-axis slices was acquired. Approximately 10 to 15 min after administration of 0.2 mmol·kg−1 gadolinium, LGE images were acquired, using a 2-dimensional segmented T1-weighted inversion recovery-prepared gradient echo sequence with optimised inversion time. An automated search through the DICOM tags identified the short-axis LGE-MRI phase-sensitive inversion recovery (PSIR) slices. These tags contain information regarding, the scanner (settings), type of scan series, patient specifics and specific about the imaging data, of which some are needed for either structuring or converting the images. Specifically, the “series description” tag was used to structure the scans into the corresponding scan modality (i.e. LGE, Cine, T1, T2 or other) and series type (i.e. 2CH, 3CH, 4CH, SAX, LAX). Subsequently, a series of DICOM slices were converted into a 3D NIfTI file, providing a standardised format for medical imaging data. It begins by sorting the DICOM slices based on their numeric prefixes in filenames, ensuring a coherent order. Metadata, such as image dimensions, pixel spacing, and slice orientation, was extracted to construct an affine transformation matrix. Then, by iterating through the slices, pixel data was read and a 3D array was populated. Finally, the resulting 3D volume is saved as a compressed NIfTI file with appropriate header information, alongside the transformation matrix.

**ECG acquisition**

The acquisition of raw 12-lead ECG signals from both sites has been described previously.^1,2^ In summary, retrospective collection of raw-format, standard 12-lead 10-second resting ECGs was conducted at both sites. Raw ECG signals were obtained from either DICOM or XML formats. A total of 77,099 ECGs were retrieved. The majority of ECGs from both sites were recorded using GE Healthcare ECG devices (approximately 70% of ECGs), with other vendors including Welch Allyn, CSYS, and DataM, each contributing roughly 10%.^1^

**Residual Variational Autoencoder Architecture**

We used a beta variational autoencoder (β-VAE) neural network to extract meaningful features from the 12-lead ECG mean waveforms and SAX LGE-MRI stacked slices.^3^ Unlike vanilla autoencoders, which are trained to encode input data, such as images or signals, into a smaller latent space for the purpose of reconstructing the original input (thus aiming to minimise the loss **L**(*x,* g(f(*x*))), where *x* as the input, f is the encoder function, and g is the decoder), VAE adds a probabilistic aspect. This allows the network to learn a probabilistic mapping between the input data and a lower-dimensional latent space. In our case, it encodes a distribution, enabling the generation of new data samples by sampling from this latent space.^4^ The reparameterisation trick was utilized to enable stochastic sampling from the learned latent space, along with Monte Carlo sampling to draw from the generative latent distribution. In our architecture (depicted in Supplementary Figure 1), convolutional layers in the encoder were used to capture spatial patterns in the (2D convolutions) ECGs and (3D convolutions) MRIs, enabling the extraction of relevant features. For the decoder, upsampling via a nearest neighbour algorithm, followed by a convolutional layer was applied to transform the sampled set of features in from the latent distribution back into the original data domain. This upsampling approach was preferred over the alternative of transposed convolutions (or deconvolutions) due to its reduced tendency to produce “checkerboard artefacts”.^5^ Each upsample layer was followed by another convolutional layer. Our VAE incorporated residual blocks, which allow for an identity mapping between activations and enable efficient information propagation across layers.^6^ In both the encoder and decoder, convolutional layers were followed by batch normalization and a Rectified Linear Unit (ReLU) activation function. The last layer in the decoder utilised a sigmoid activation function, mapping the output to values within the range [0, 1]. The β-VAE learns by optimizing the Evidence Lower Bound (ELBO), which can be formulated as:

$$\mathrm{ELBO}=\mathbb{E}_{q(z|x)}[\log p(x|z)]- \beta\cdot KL(q(z|x)||p\left( z \right))$$

This objective comprises two key components: the expected log-likelihood $\mathbb{E}_{q(z|x)}\left[ \log p\left( x | z \right) \right]$, signifying the reconstruction loss, and the *β*-weighted Kullback-Leibler (KL) divergence $KL(q(z|x)||p\left( z \right))$, which imposes a regularisation on the latent space distribution. The β-VAE learns by optimising the loss function, which is a sum of the reconstruction loss given by the expected log-likelihood and a regularising KL-divergence which measures the divergence between the learned latent variable distribution *q*(*z*|*x*) and some prior distribution (Gaussian for our model) *p*(*z*), with β controlling the balance between these two aspects. Therefore, the KL divergence acts as a regulariser by enforcing the latent space to approximate a standard Gaussian distribution. We used the Adam optimiser with mini-batch sizes of 32, and a learning rate scheduler starting from 1e-4 (ECG-VAE) and 1e-5 (CMR-VAE).^7^ The learning rate was reduced by a factor of 10 upon encountering an error plateau. The weights of the VAEs were initialised using Xavier initialization method.^8^ The ResNet VAEs were developed using Pytorch (version 2.0.5).

**β parameter tuning**

The β parameter plays a crucial role in forcing the model to learn disentangled presentations^9^, and managing the trade-off between the model's performance in terms of reconstruction accuracy and the quality of the learned latent space.^3,10^ Various experiments were conducted to determine the optimal β parameter values that balance disentanglement and reconstruction loss. We tuned the optimal β parameter value for both VAEs, learning curves are in Supplementary Figures 2-3.

**Model training**

The 2D-ECG VAE model was trained on a total of 333,304 12-lead ECGs, encoding to a latent space dimensionality of 32, to reach an initial state where the weights resonate with the common domain of ECG signals morphologies. From these, 256,205 ECGs were derived from an ECG database consisted of routine ECGs collected at the general ward and outpatient clinic of the Amsterdam Medical Center between 1998 and 2018.28 These ECGs were extracted from the MUSEweb data management system (GE Healthcare, Chicago, Illinois, United States of America). The database includes one ECG per patient (mean age of 50 years, range 18–60 years, 52% male). An additional 77,099 12-lead ECGs were from an ICD registry, of which details have been published.^1,2^ Similarly, the 3D-MRI VAE model was trained on 970 short-axis LGE-MRIs from the registry^1,2^, encoding scans to a latent space dimension of 256.

**Reconstructive performance assessment**

The evaluation of the reconstructive performance of the 2D-ECG ResNet VAE model involved the computation of the Pearson’s correlation coefficient (PCC), root mean squared error (RMSE), and percentage root mean square difference (PRD), as provided by Beetz et al. (2022)^11^, between the original and reconstructed signals. PCC measures the linear correlation between the original and reconstructed ECG signals, with a value nearing 1 indicating a perfect positive correlation. RMSE quantifies the average magnitude of differences between the original and reconstructed signals, where smaller values signify better reconstructive performance. PRD, akin to RMSE, provides a relative and normalised quantification of the reconstructions.

The assessment of the MRI reconstructions as provided by the 3D-MRI ResNet VAE model was according to the structural similarity index measure (SSIM), peak-to-noise ratio (PNR), mutual information (MI), and normalized RMSE (NRMSE). SSIM measures the structural similarity between the original and reconstructed images, with values ranging from -1 to 1 and 1 denoting perfect similarity. PNR quantifies the peak signal-to-noise ratio between the original and reconstructed slices, with higher PNR values indicating superior quality in the compressed or reconstructed image. MI is a metric derived from the joint (2D) histogram, with high values suggesting a concentrated signal in few bins and low values indicating a dispersed signal across many bins. NRMSE provides a relative measure of error, with smaller values indicating enhanced performance.

**Extreme Gradient Boosting Algortihm (XGBoost) developement**

We applied the extreme gradient boosting (XGBoost) algorithm for binary classification of the endpoint. XGBoost is a decision tree-based ensemble machine learning technique. Model performance was evaluated using the logloss (negative log-likelihood) as the loss function for binary classification. Optimal hyperparameters, including the learning rate and those defining the depth and structure of the tree-based architecture, were selected using a Bayesian optimisation technique using the HyperOpt library (version 0.2.7) for Python.^12^ The hyperparameter space that we used during optimisation is provided as a Supplementary Table.

**Latent representation traversal**

Latent traversal involves systematically adjusting the latent vector, which represents the learned features in the latent space, for a given instance. The mean and standard deviation of each latent variable are calculated across all subjects. The latent vector is then modified for each latent variable: subtracting three standard deviations for the lower bound, using the mean latent value, and adding three standard deviations for the upper bound. This traversal is performed for each factor, offering insights into how the VAE responds to variations in these factors. Factors could represent different features or characteristics in the input data. To create a 3D geometric representation from individual slices, linear interpolation is applied, ensuring a consistent number of slices across different factors. The absolute differences between pixel values in the lower and upper bounds are calculated and normalized. The results are presented as a 3D surface map, providing a visual understanding of how variations in latent variables influence the reconstructed data. Additionally, ECG reconstructions are smoothed using a moving average and displayed per lead, offering a clearer representation of the reconstructed ECG signals.

**Gradient-based activation mapping**

We extracted and visualised attention maps from the convolutional layers of the VAE networks for MRI and ECG input data. This approach has been previously described.^13^ The implementation included the computation of gradients through backward propagation, and the convolutional layer outputs during the forward pass for the same target layer. These were combined to latent representation-specific attention maps. We calculated attention maps through encoding of one-hot batches. A Rectified Linear Unit (ReLU) activation was applied to the attention map. In case the pooled effect of multiple latent representations was evaluated, these maps were averaged and normalized. The resulting attention maps are superimposed on the original MRI slices and 12-lead ECG, highlighting regions of interest.

# Supplementary Tables

**Supplementary table 1.** Percentages of missing values in the clinical patient data

| **Variable** | **Missing percentage (%)** |
| --- | --- |
| **Demographics** |  |
| Age | 0.0 |
| Gender | 0.0 |
| Body Mass Index | 6.6 |
|  |  |
| **Medical history** |  |
| Atrial Arrhythmia | 0.0 |
| Non-Sustained Ventricular Tachycardia | 0.0 |
| Hypertension | 0.0 |
| QRS Duration | 1.0 |
| Cerebrovascular Accident | 0.0 |
| Chronic Obstructive Pulmonary Disease | 0.0 |
| Diabetes Mellitus | 0.0 |
| Underlying pathology | 0.0 |
| Left Ventricular Ejection Fraction | 0.0 |
| Peripheral Artery disease | 0.0 |
| Congenital Heart Disease | 0.0 |
| Dyslipidemia | 0.3 |
|  |  |
| **Laboratory Values** |  |
| Sodium | 19.4 |
| Potassium | 18.3 |
| Creatinine | 12.1 |
|  |  |
| **Medication** |  |
| Aldosterone Antagonist | 5.5 |
| Vitamin K Antagonist | 5.5 |
| Antiarrhythmic Type: Sotalol | 5.5 |
| Antiarrhythmic Type: Digoxin | 5.5 |
| Antiarrhythmic Type: Amiodarone | 5.5 |
| β-Blocker | 5.2 |
| NOAC | 5.5 |
| Diuretics | 5.5 |
| Angiotensin Receptor Blocker | 5.5 |
|  |  |
| **Implanted device** | 0.0 |

**Supplementary table 2.** Search space used for tuning of hyperparameters of the extreme gradient boosting algorithm (XGBoost)

| **Hyperparameter** | **Lower Bound** | **Upper Bound** |
| --- | --- | --- |
| Learning Rate | 0.0001 | 0.01 |
| Number of Estimators | 100 | 500 |
| Maximum Tree Depth | 3 | 8 |
| Column Sampling Rate | 0.3 | 0.9 |
| Subsampling Rate | 0.7 | 1.0 |
| Minimum Child Weight | 1 | 5 |
| Class Weighting | 9 | 10 |

**Supplementary table 3.** Clinical variables used for predictive modelling

| **Category** | **Variable name** |
| --- | --- |
| Demographics | Age |
|  | Sex |
|  | Body Mass Index |
| Laboratory values | Sodium |
|  | Potassium |
|  | Creatinine |
| Cardiomyopathy | Hypertrophic |
|  | Dilated |
|  | Genetic |
|  | Miscellaneous |
| Medical history | Hypertension |
|  | Diabetes mellitus |
|  | COPD |
|  | Cerebrovascular accident |
|  | Atrial arrhythmia |
|  | NSVT |
|  | Peripheral artery disease |
|  | Congenital heart disease |
|  | Dyslipidaemia |
|  | QRS duration |
| Medication | Vitamin K antagonist |
|  | Aldosterone antagonist |
|  | Sotalol |
|  | Digoxin |
|  | Amiodarone |
|  | β-Blocker |
|  | Novel Oral Anticoagulant |

**Supplementary table 4.** Reconstruction performances of the 3D ResNet VAE

| **Slice reconstruction** | | **Mean ±SD** |
| --- | --- | --- |
|  | Structural similarity index | 0.3929 ±0.0946 |
|  | Peak signal-to-noise ratio | 17.0809 ±0.9755 |
|  | Mean squared error | 0.0158 ±0.0050 |
|  | Normalised Root Mean Square Error | 0.2284 ±0.0422 |
|  | Mutual information | 0.7346 ±0.197 |
| **Myocardium mask** | |  |
|  | Structural similarity index | 0.5928 ±0.0984 |
|  | Peak signal-to-noise ratio | 19.4177 ±1.0083 |
|  | Mean squared error | 0.0085 ±0.0023 |
|  | Normalised Root Mean Square Error | 0.2964 ±0.0604 |
|  | Mutual information | 0.8714 ±0.198 |

**Supplementary table 5.** Reconstruction performances of the 2D ResNet VAE for ECG reconstruction

|  | **ECGs** | **Model architecture** | **Root Mean Squared Error** | **Percentage Root Mean Squared Difference** | **Pearson correlation coefficient** |
| --- | --- | --- | --- | --- | --- |
| Beetz et al. (2022) ^11^ | 1300 median beats lead II | 2D CNN-VAE | 0.16 | 26.51 | N/A |
| Zhu et al. (2019) ^14^ | 48 single lead | BiLSTM-CNN GAN | 0.28 | 66.41 | N/A |
| Van der Leur et al. (2022) ^15^ | 1,144,331 median beats 12-lead | β-VAE | N/A | N/A | 0.90 |
| Delaney et al. (2019) ^16^ | 46 single lead 10-second ECG | 1CNN BiLSTM GAN | N/A | N/A | N/A |
| Kolk et al. (2023)^1^ | Pre-train 256,205 / Fine-tuning cohort 77,099 mean 6-lead ECG waveforms | β-VAE | 0.050 ±0.026 | 9.49 ±5.03 | 0.93 ±0.09 |
| Current study | 333,304 mean 12-lead ECG waveforms | ResNet 2D CNN-VAE | 0.036 ±0.003 | 5.81 ±0.82 | 0.97 ±0.005 |

**Supplementary table 6.** Performance on the external validation test for one-year appropriate ICD-therapy risk prediction

|  | **DEEP RISK** | **Clinical branch** | **ECG branch** | **MRI branch** |
| --- | --- | --- | --- | --- |
| Accuracy | 0.741 (0.602, 0.961) | 0.625 (0.214, 0.922) | 0.643 (0.136, 0.864) | 0.706 (0.515, 0.951) |
| F1-score | 0.324 (0.128, 0.667) | 0.220 (0.069, 0.455) | 0.207 (0.066, 0.400) | 0.290 (0.105, 0.588) |
| Sensitivity | 0.981 (0.75,0 1.000) | 0.819 (0.364, 1.000) | 0.703 (0.333, 1.000) | 0.955 (0.714, 1.000) |
| Specificity | 0.726 (0.580, 0.969) | 0.613 (0.172, 0.938) | 0.641 (0.091, 0.878) | 0.690 (0.495, 0.96) |
| PPV | 0.206 (0.068, 0.556) | 0.138 (0.036, 0.364) | 0.130 (0.034, 0.280) | 0.181 (0.056, 0.500) |
| NPV | 0.999 (0.986, 1.000) | 0.984 (0.944, 1.000) | 0.975 (0.930, 1.000) | 0.996 (0.976, 1.000) |
| AUROC | 0.844 (0.713, 0.961) | 0.639 (0.386, 0.867) | 0.544 (0.26, 0.816) | 0.801 (0.649, 0.936) |
| AUPRC | 0.313 (0.083, 0.652) | 0.131 (0.035, 0.294) | 0.099 (0.033, 0.206) | 0.232 (0.061, 0.499) |
| *Abbreviations*: AUROC=Area Under the Receiver Operating Characteristic curve, AUPRC=Area Under the Precision-Recall Curve,  ECG=: Electrocardiogram, PPV=Positive Predictive Value, MRI=Magnetic Resonance Imaging, NPV=Negative Predictive Value | | | | |

# Supplementary Figures

**Supplementary figure 1.** Schematic overview of the ResNet VAEs for MRI (3D) and ECG (2D) reconstruction

**
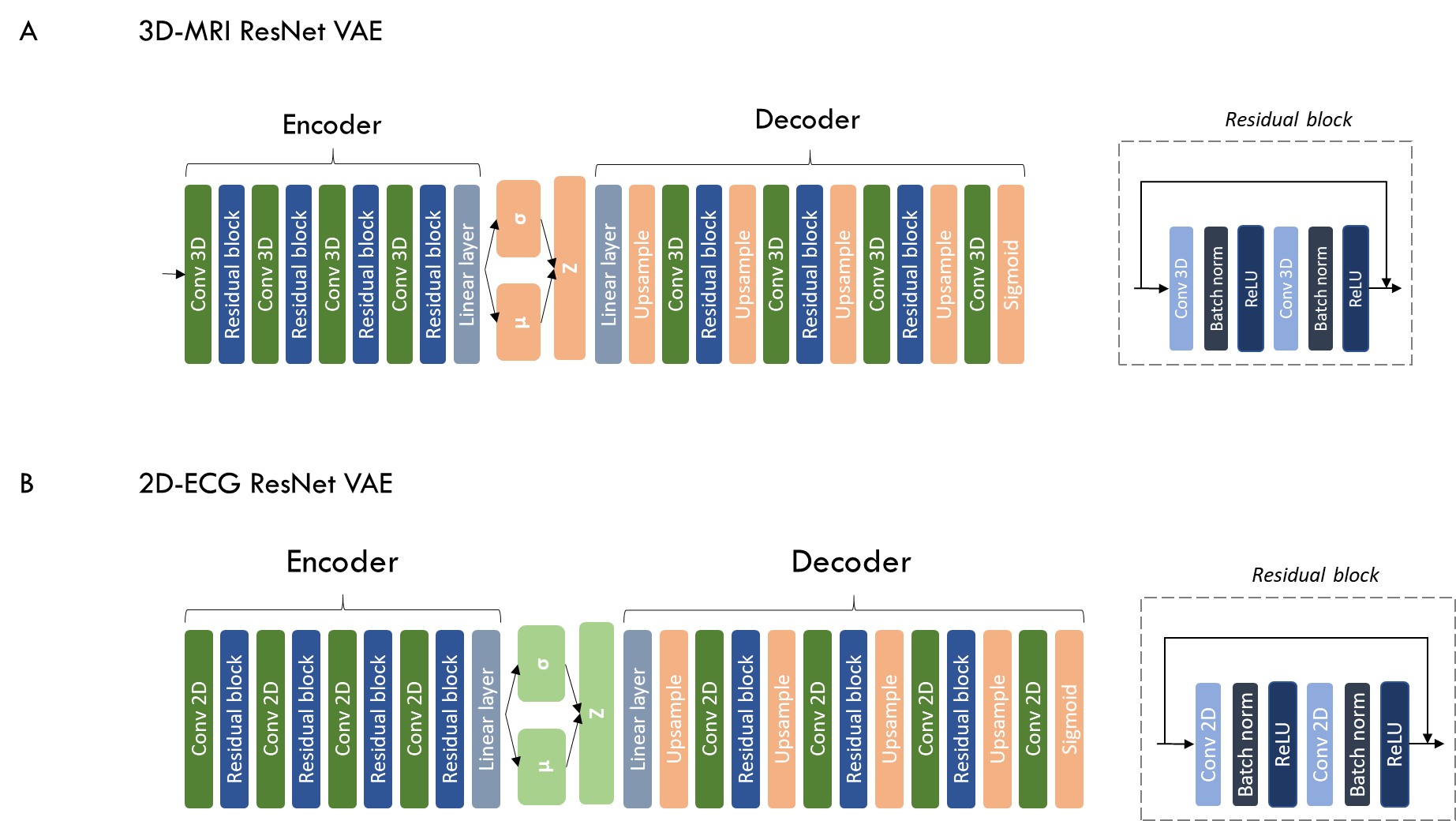
**

**Supplementary figure 2.** Model development and extraction of the latent representations from patients in the NICM cohort

**
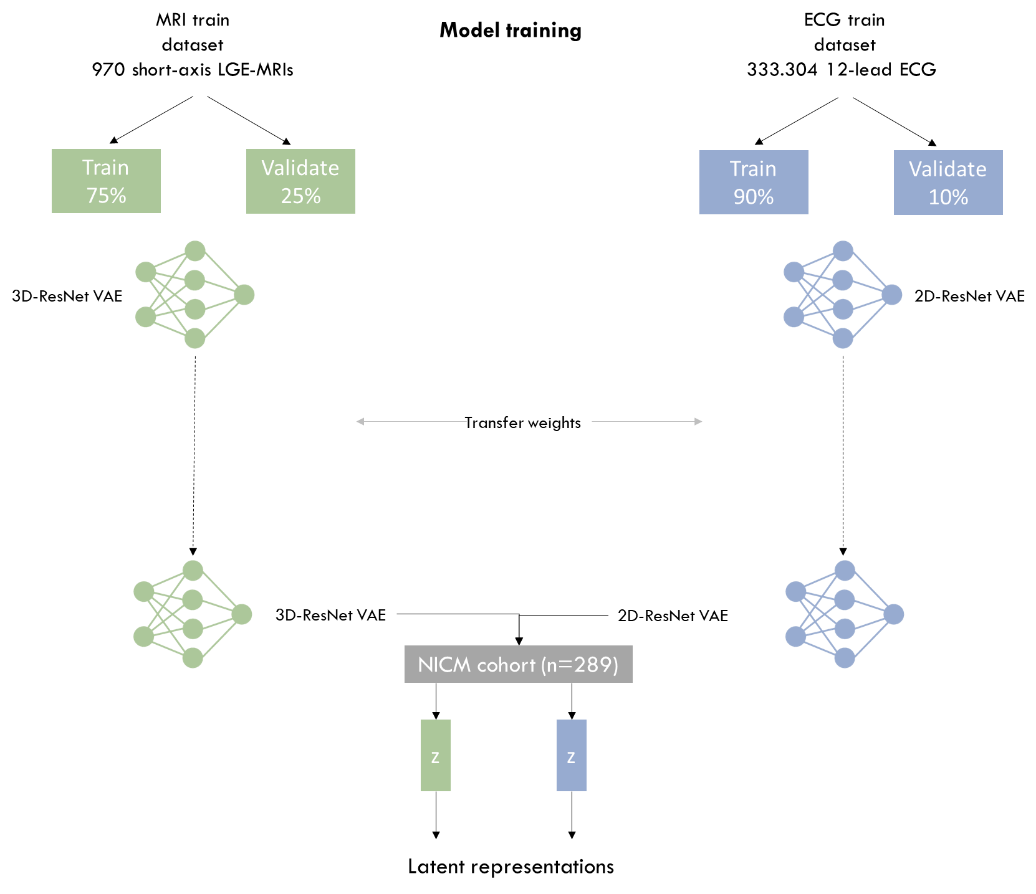
**

**Supplementary figure 3.** Optimisation of the β-parameter of the ResNet variational autoencoder (VAE) for 12-lead ECG reconstruction


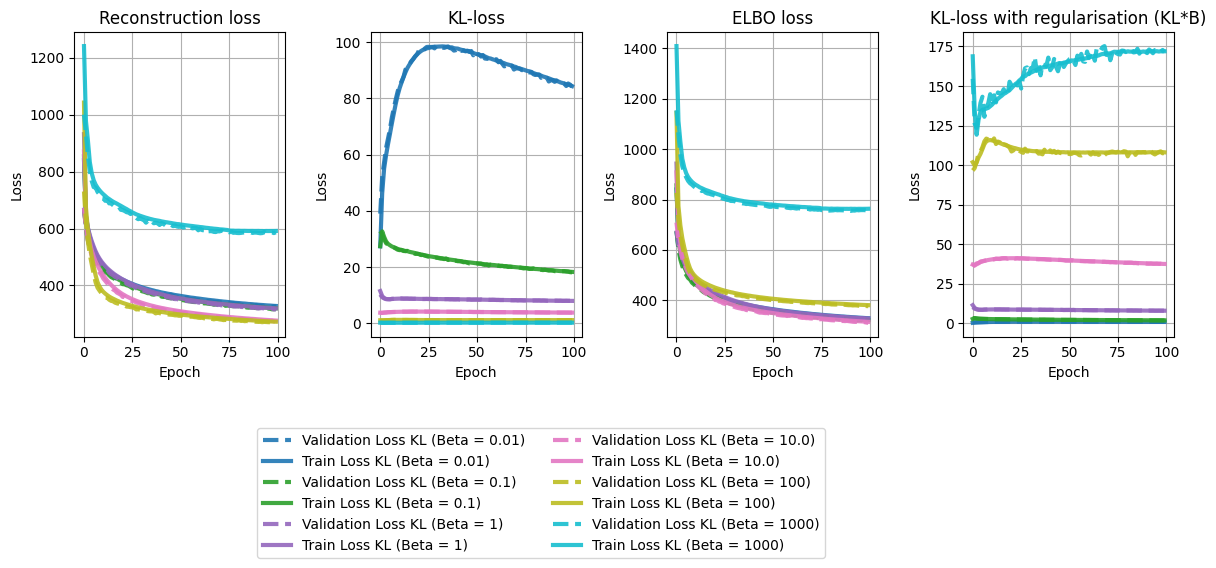


**Supplementary figure 4.** Optimisation of the β-parameter of the ResNet variational autoencoder (VAE) for short-axis LGE-MRI reconstruction


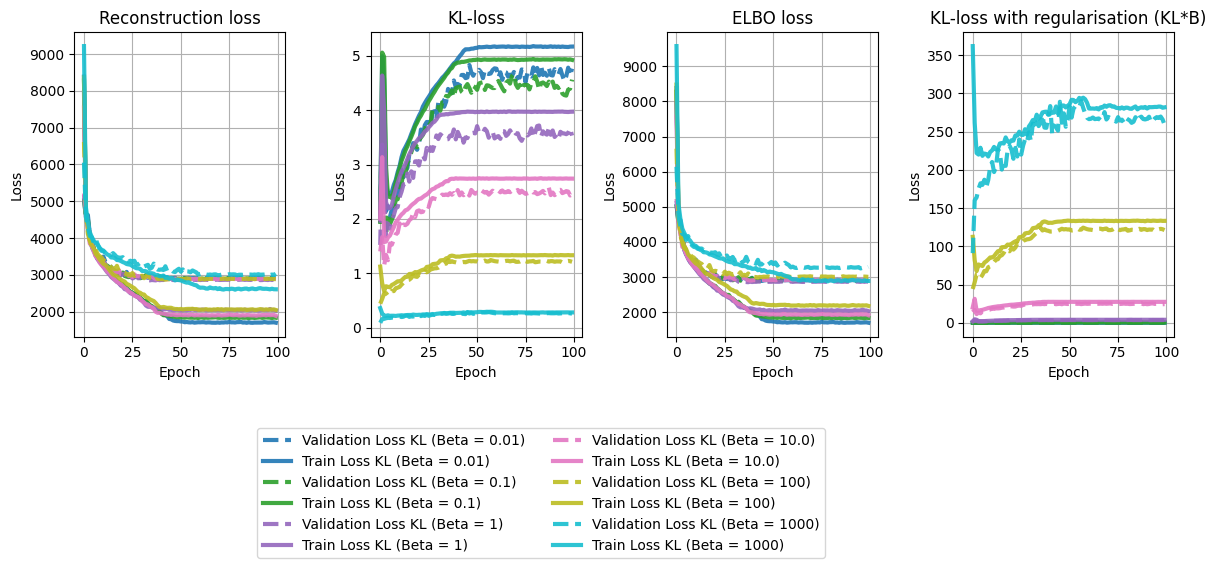


**Supplementary figure 5.** CONSORT diagram of patient selection


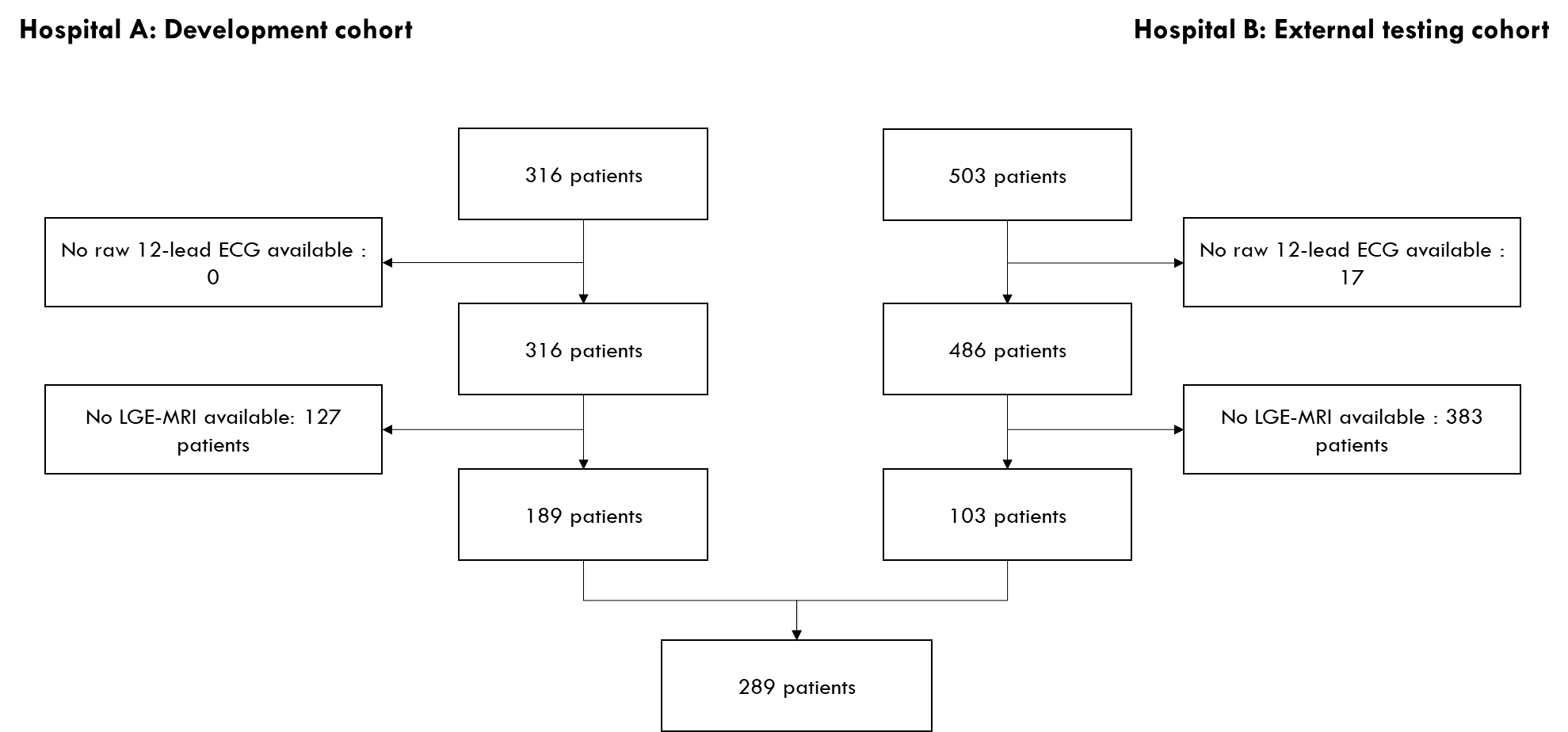


**Supplementary figure 6.** Original and reconstructed mean 12-lead ECG waveforms


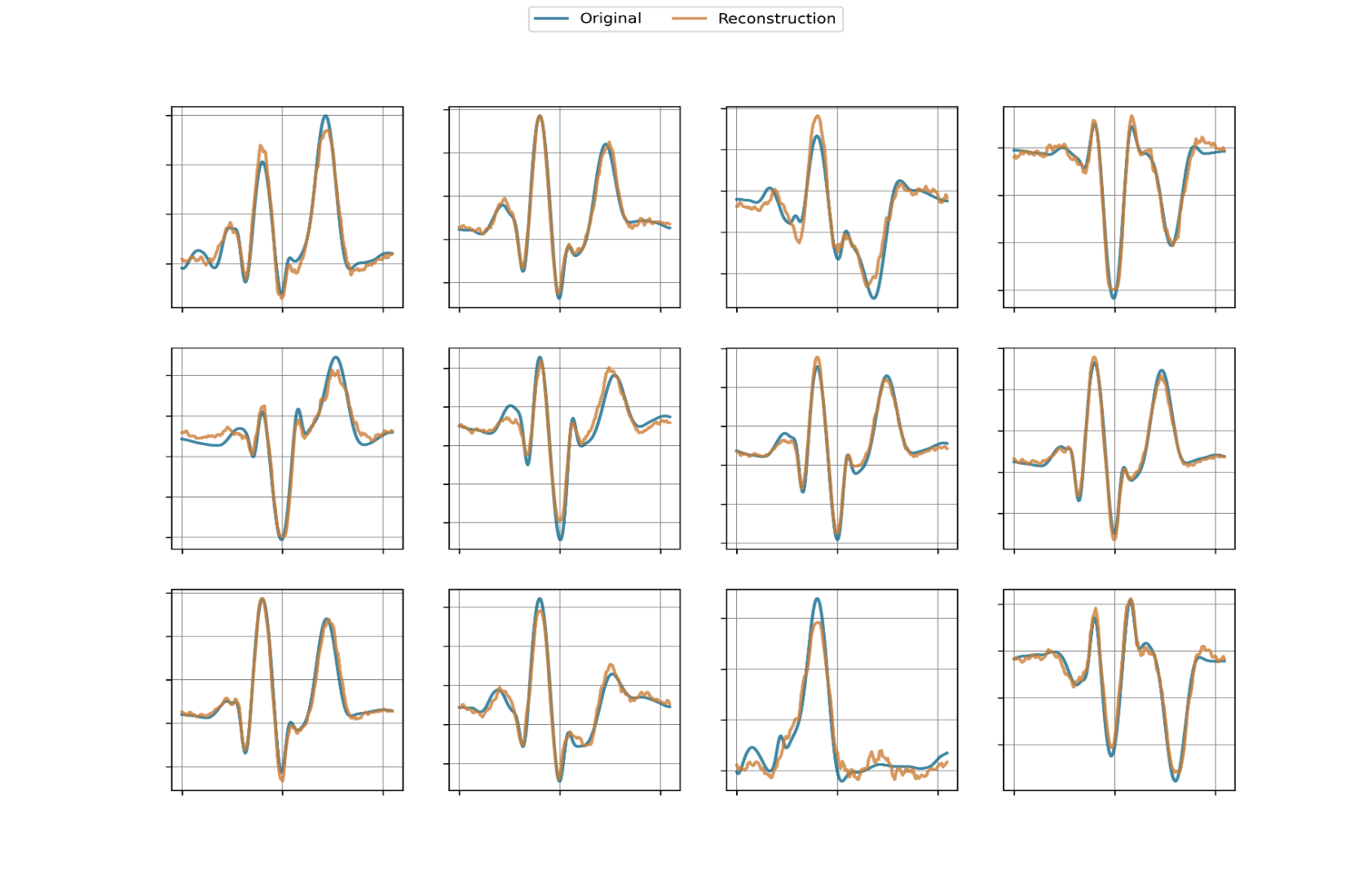


**Supplementary figure 7.** Original and reconstructed short-axis LGE-MRI images

**
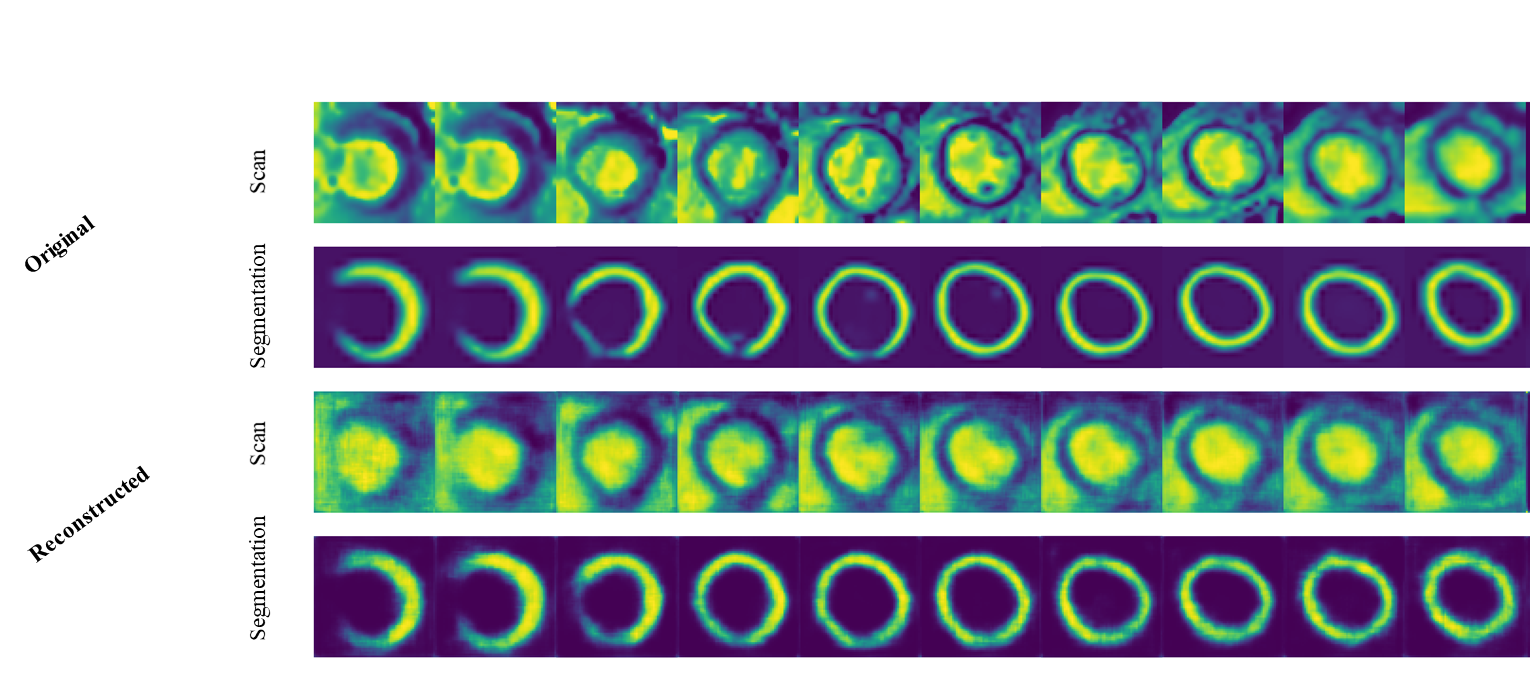
**

**Supplementary figure 8.** Calibration curve for the multimodal prediction model


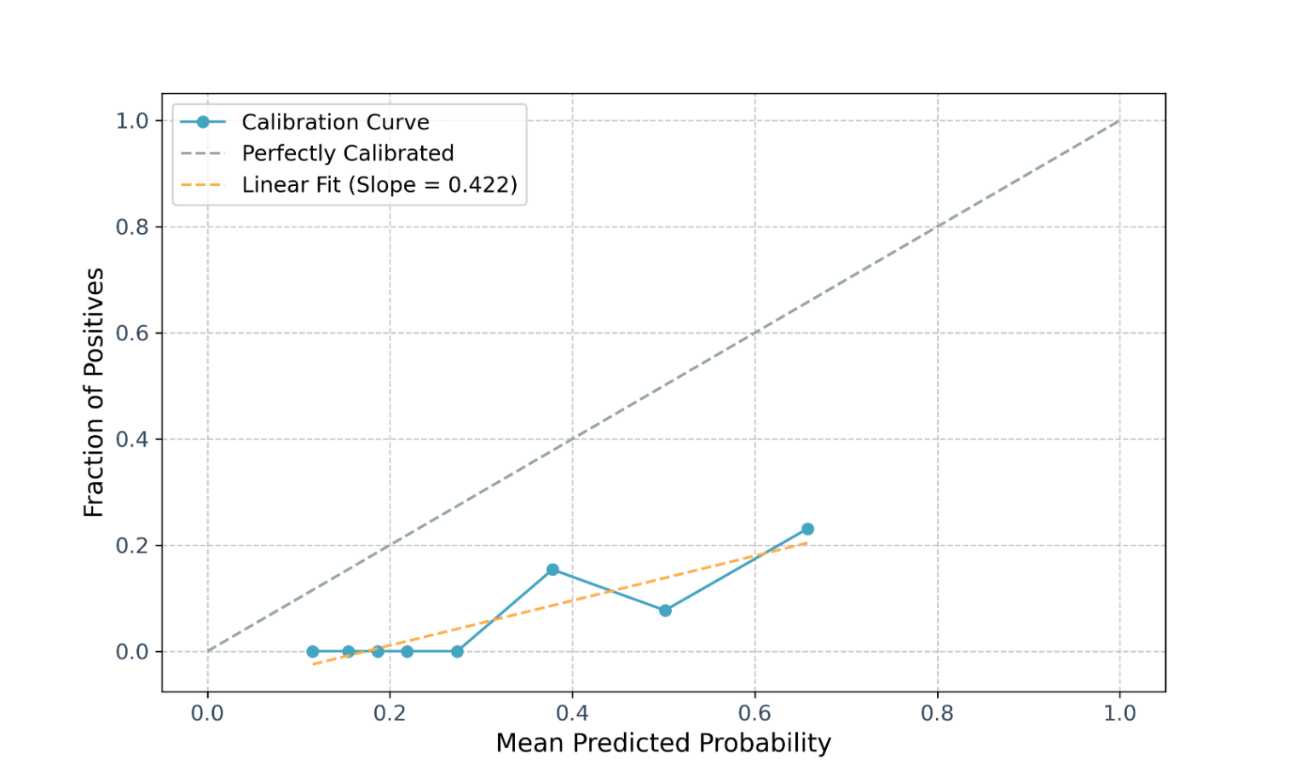


**References**

1. Kolk MZH, Ruiperez-Campillo S, Alvarez-Florez L, et al. Dynamic prediction of malignant ventricular arrhythmias using neural networks in patients with an implantable cardioverter-defibrillator. *EBioMedicine* 2023; **99**: 104937.

2. Kolk MZH, Ruiperez-Campillo S, Deb B, et al. Optimizing patient selection for primary prevention implantable cardioverter-defibrillator implantation: utilizing multimodal machine learning to assess risk of implantable cardioverter-defibrillator non-benefit. *Europace* 2023; **25**(9).

3. Diederik PK, Max W. Auto-Encoding Variational Bayes. *CoRR* 2013; **abs/1312.6114**.

4. Diederik PK, Tim S, Max W. Improved Variational Inference with Inverse Autoregressive Flow. *ArXiv* 2016; **abs/1606.04934**.

5. Odena A, Dumoulin V, Olah C. Deconvolution and checkerboard artifacts. *Distill* 2016; **1**(10): e3.

6. Kaiming H, Zhang X, Shaoqing R, Jian S. Deep Residual Learning for Image Recognition. *2016 IEEE Conference on Computer Vision and Pattern Recognition (CVPR)* 2015: 770-8.

7. Kingma DP, Ba J. Adam: A Method for Stochastic Optimization. *CoRR* 2014; **abs/1412.6980**.

8. Glorot X, Bengio Y. Understanding the difficulty of training deep feedforward neural networks. International Conference on Artificial Intelligence and Statistics; 2010; 2010.

9. Christopher PB, Irina H, Arka P, Loc M, Nicholas W, Guillaume D, Alexander L. Understanding disentangling in $\beta$-VAE. *ArXiv* 2018; **abs/1804.03599**.

10. Higgins I, Matthey L, Pal A, et al. beta-VAE: Learning Basic Visual Concepts with a Constrained Variational Framework. International Conference on Learning Representations; 2016; 2016.

11. Beetz M, Banerjee A, Grau V. Multi-Domain Variational Autoencoders for Combined Modeling of MRI-Based Biventricular Anatomy and ECG-Based Cardiac Electrophysiology. *Front Physiol* 2022; **13**: 886723.

12. Frazier P. A Tutorial on Bayesian Optimization. *ArXiv* 2018; **abs/1807.02811**.

13. Liu W, Li R, Zheng M, et al. Towards Visually Explaining Variational Autoencoders. *2020 IEEE/CVF Conference on Computer Vision and Pattern Recognition (CVPR)* 2019: 8639-48.

14. Zhu F, Ye F, Fu Y, Liu Q, Shen B. Electrocardiogram generation with a bidirectional LSTM-CNN generative adversarial network. *Sci Rep* 2019; **9**(1): 6734.

15. van de Leur RR, Bos MN, Taha K, et al. Improving explainability of deep neural network-based electrocardiogram interpretation using variational auto-encoders(). *Eur Heart J Digit Health* 2022; **3**(3): 390-404.

16. Delaney AM BE, Ward TE. Synthesis of Realistic ECG using Generative Adversarial Networks. *arXiv* 2019; **1909**.
